# Aberrations of DNA Repair Pathways in Prostate Cancer: Future Implications for Clinical Practice?

**DOI:** 10.3389/fcell.2018.00071

**Published:** 2018-09-05

**Authors:** Orazio Caffo, Antonello Veccia, Stefania Kinspergher, Mimma Rizzo, Francesca Maines

**Affiliations:** Department of Medical Oncology, Santa Chiara Hospital, Trento, Italy

**Keywords:** prostate cancer, PARP inhibitors, BRCA1, BRCA2, olaparib

## Abstract

Patients who are carriers of inherited mutations in essential component of DNA repair pathways have a significantly higher lifetime risk for developing cancer compared to the population of reference. Recent advances in DNA next-generation sequencing technology have allowed screening for carriers of those mutations, allowing development of promising risk-reduction strategies and providing the rationale to personalize the therapeutic approach for these patients. New intriguing scenarios are opening nowadays for the management of prostate cancer in patients with germline or somatic mutations in components of DNA repair pathways (e.g., *BRCA1* and *BRCA2* genes), such as specific screening policies and new therapeutic strategies involving PARP inhibitors or platinum-based chemotherapy.

## Introduction

*BRCA1* and *BRCA2* are tumor suppressor genes with essential functions in the maintenance of genome stability (Yoshida and Miki, 2004). Both genes are characterized by an autosomal dominant inheritance pattern with incomplete penetrance, and individuals with heterozygous germline mutations in BRCA1/2 genes are at risk of losing the functional allele as a consequence of a second damage induced by alkylating agents, ionizing radiation, reactive oxygen species or chemical mutagens (Evers et al., 2010). The functional loss of *BRCA1 or 2* leads to a defect in double-strand breaks repair through the homologous recombination process, which, in turn, drastically affects the ability of the cell to preserve genome fidelity and stability (Liu and West, 2002).

It is well established that women who are carriers of inherited harmful *BRCA 1* or *2* gene mutations have an increased risk to develop breast and/or ovarian cancer in lifetime compared to their wild-type counterparts (Easton et al., 2007). Importantly, these cancer susceptibility genes have been associated to an increased risk of developing several other types of tumor, such as fallopian tube and peritoneal cancer in women (Brose et al., 2002; Finch et al., 2006), breast and prostate cancer (PC) in men (Levy-Lahad and Friedman, 2007; Tai et al., 2007; Mersch et al., 2015) and pancreatic cancer in both sexes (Ferrone et al., 2009; Mersch et al., 2015) although not fully overlapping results were observed (van Asperen et al., 2005; Moran et al., 2012).

## BRCA and prostate cancer

The incidence of germline BRCA mutations in newly diagnosed PC, unselected for family predisposition, ranges approximately from 1.2 to 2% of the cases (Leongamornlert et al., 2012). BRCA carriers have an increased risk to develop PC (at 1.8- to 4.5-fold for BRCA1 carriers and at 2.5- to 8.6-fold for BRCA2 carriers in patients aged <65 years), in particular at early onset (Kote-Jarai et al., 2011; Leongamornlert et al., 2012).

Management of high-risk men with germline mutations in DNA-repair genes is uncertain and controversial, without consensus on the screening for PC. In this population, the harms of overdiagnosis and overtreatment are mitigated by the increased incidence and risk of PC-specific mortality. The National Comprehensive Cancer Network guidelines recommend that men with BRCA mutations should perform breast self-examination starting at 35 years of age and annually thereafter. At 45 years of age BRCA2 carriers should begin PSA screening and BRCA1 carriers should be advised to consider it. Prostatic biopsy is recommended at PSA > 3.0 ng/mL in this population [15].

The IMPACT study (**I**dentification of **M**en with a genetic predisposition to **P**rost**A**te **C**ancer: **T**argeted screening in BRCA1/2 mutation carriers and controls) evaluated a tailored PC screening in 1522 men with BRCA1/2 germline mutation, proposing annual PSA tests and a prostate biopsy if PSA >3 ng/mL (Bancroft et al., 2014). Despite the lack of statistically significant difference in PC detection rate between carriers and controls, the authors observed a higher incidence of PC in BRCA2 carriers compared to BRCA1 and controls (3.3 vs. 2.6% and <2%, respectively); in addition more than 2/3 of the PCs detected in the BRCA2 carriers and 61% in BRCA1 carriers were classified as intermediate or high risk. In light of these results, they suggested that mainly in BRCA2 carriers PSA testing should be proposed earlier, repeated at shorter screening intervals, and with lower PSA thresholds compared to the general population, in order to detect the tumorigenic transformation earlier. It is clear that according to the actual limitations of PSA-based screening, final results of the study are waiting to define the optimal screening program in this subset of patient, although the preliminary results seem to support PSA routine testing in BRCA carriers (Mitra et al., 2011).

An ongoing clinical trial in Toronto, Canada (NCT01990521) is evaluating the role of prostate MRI in male BRCA carriers, regardless of the PSA values.

BRCA2 germline mutations in men with PC have been associated with an aggressive tumor phenotype, a more advanced tumor stage at the diagnosis and poor survival outcomes at any disease stage, including the localized/locally advanced disease (Tryggvadottir et al., 2007; Castro et al., 2013). In particular, it has been recently observed that BRCA mutation carriers with localized PC have worse outcomes than those who are wild type regardless of the local treatment they have previously undergone (radical prostatectomy or radiation therapy; Castro et al., 2015). The 5-year metastasis-free survival was significantly higher in wild-type patients compared to mutation carriers (93 vs. 77%; *p* = 0.009). BRCA carriers had higher rate of lymph-nodes involvement, higher Gleason score, developed distant metastasis earlier and had a shorter survival. Overall, the independent prognostic value of this mutation at the multivariate analysis strongly suggests the need of a timely management in BRCA carriers (Castro et al., 2013, 2015). Furthermore, these patients developed more frequently castration-resistant PC (CRPC) upon occurrence of metastases (Castro et al., 2013).

Overall, several studies suggested that BRCA mutation is an independent negative prognostic factor for both overall survival and PC-specific survival (Modena et al., 2016).

Taken together, all these findings suggest that active surveillance may not be a valid treatment option for BRCA mutation carriers, even in the low-risk PC population, according to more aggressive behavior and poor disease outcomes observed in such subjects. For all these reasons screening for BRCA1/2 might be useful in early diagnosis and potentially have a beneficial impact on the management of these patients.

Aberrations in genes involved in DNA integrity seem to increase in the late-stages of PC disease with 20-30% men with metastatic CRPC (mCRPC) carrying genomic defects in DNA-repair pathways (Mateo et al., 2017). At the moment it is not clear if the increased incidence of defects in DNA-repair in mCRPC is related to progression to a more aggressive disease phenotype or rather it is the result of a secondary pressure due to specific treatments.

## Implications for the treatment

In the last decades the therapeutic landscape of mCRPC patients has dramatically changed due to availability of several agents able to significantly improve survival: chemotherapeutic agents, docetaxel (Tannock et al., 2004) and cabazitaxel (de Bono et al., 2010), new generation hormone agents, abiraterone (de Bono et al., 2011; Ryan et al., 2014), and enzalutamide (Scher et al., 2012; Beer et al., 2014), and one radiopharmacetical agent, radium 223 (Parker et al., 2013).

In addition, there is a growing interest for strategies based on the immunotherapy: after the studies on vaccines leading to the FDA approval of sipuleucel-T (Kantoff et al., 2010a) or to the development of PROSTVAC-VF (Kantoff et al., 2010b), immune-checkpoints inhibitors are currently being tested in mCRPC, such as pembrolizumab (NCT02787005) and atezolizumab in combination with enzalutamide (NCT03016312).

The use of targeted therapies such as poly-(ADP-ribose) polymerase (PARP) inhibitors in BRCA-associated breast and ovarian cancers (Audeh et al., 2010; Tutt et al., 2010; Ledermann et al., 2012) suggests a potential role of these drugs also in BRCA carriers affected by other solid tumors, including PC. PARP polymerase is a nuclear DNA-binding enzyme involved in the single-strand break DNA repair, through the base excision and repair (BER) pathway (Morales et al., 2014). Impairment of BER activity through PARP inhibition determines the so called *synthetic lethality* interaction in homologous recombination deficient BRCA-mutant cancer cells (“BRCA-ness”), an overwhelming genome instability condition which drives cancer cells to die (Farmer et al., 2005). BRCAness tumors seem to be highly sensitive to PARP inhibitors, independently of the site of origin of the tumor (Underhill et al., 2011).

Several PARP inhibitors (olaparib, rucaparib, niraparib, velaparib, and talazoparib) are currently being investigated in several tumor types. In the case of BRCAness PCs, phase I clinical trials (Fong et al., 2009; Sandhu et al., 2013a), small mCRPC series (Sandhu et al., 2013b), and translational studies (Brenner et al., 2011) suggested a role for these agents also in PC patients. Orally administered Olaparib at 400 mg twice a day was tested in 298 patients with germline BRCA1/2 mutation and recurrent advanced cancers, including eight pre-treated PC patients (Kaufman et al., 2015). Among these PC patients, seven had a BRCA2 mutation and the remaining was a BRCA1 carrier. All these patients had previously received an average of two lines of treatment for the advanced disease. Although the very limited number of PC patients, the results were encouraging since 4 out of 8 patients showed tumor response while in 2 the disease remained stable. The median progression-free survival (PFS) was 7.2 months with two patients having a favorable response for longer than 1 year. It is noteworthy that only one out of four PC patients who had previously received platinum chemotherapy responded to the PARP inhibitor, suggesting a potential cross-resistance between the mechanisms of action of these drugs (Kaufman et al., 2015).

On the basis of these promising results, the study TOPARP-A (a larger phase II clinical trial) investigated the activity of Olaparib in 50 mCRPC patients who had shown progression disease after one or two treatments, including docetaxel (Mateo et al., 2015). In this study genomic defects in DNA-repair genes were prospectively evaluated with next-generation sequencing analyses on fresh tumor-biopsy performed before the treatment. The primary endpoint of the study was composite: radiological response according to RECIST 1.1 and/or PSA declines >50% and/or conversion in circulating tumor cells (CTC). PFS and OS were secondary endpoints of the study. A response to Olaparib was observed in 16 patients (33%), who received the drug for 6 months in 12 cases and for 12 months in four cases. The median OS was 10.1 months (5.1–15.6). Molecular analyses identified aberrations in DNA-repair genes [BRCA 1/2, ataxia-telangiectasia mutated (ATM), Fanconi's anemia genes, CHEK2, PALB2, FANCA, HDCA2, and others] in 16/49 patients. Of these 16 biomarker-positive patients, 14/16 (88%) showed a response to Olaparib, of particular relevance, 7/7 patients with BRCA2 mutation and 4/5 patients with ATM aberrations. The PSA response in those who had a clinical benefit from PARP inhibitors (13/16, 81%) suggested that PSA monitoring during the treatment could be useful to rapidly identify the responders. Radiologic PFS and OS were significantly longer in the biomarker-positive compared to the biomarker-negative group (median 9.8 vs. 2.7 months, *p* < 0.0001; median 13.8 vs. 7.5 months, *p* = 0.05, respectively; Mateo et al., 2015).

The tolerability profile of the drug was manageable and mainly related to hematological toxicities (anemia, thrombocytopenia), fatigue and gastrointestinal side effects. The striking results in BRCA1/2 and ATM gene-mutated mCRPC patients led the US Food and Drug Administration (FDA) to approve this breakthrough therapy with Olaparib for this population, although efficacy and safety results of the phase II trial need to be confirmed in larger trials. Noteworthy, it could be reductive to restrict treatment with Olaparib to patients with BRCA or ATM mutations only since this drug showed to be effective in additional 25% of patients who are very likely carriers of unknown defects in homologous recombination.

The Part B of TOPARP study (NCT01682772) (TOPARP-B) aims to validate the role of Olaparib in BRCA2 or ATM carriers and to provide additional efficacy data in presence of less common mutations in other genes involved in DNA repair such as FANC, CDK12, RAD51, PALB2, ATR, CHEK1, CHEK2, DSS1, MRE11, XRCC2/3, and ETS gene fusions (TMPRSS2-ERG) which have been previously linked to PARP inhibitors sensitivity (McCabe et al., 2006; Yang et al., 2011; Hussain et al., 2014).

Additional studies are waiting to confirm that the frequency of DNA-repair defects in mCRPC patients is higher than that observed in other disease settings or in untreated patients. Preliminary results suggest that somatic BRCA mutations are more often observed in late stages of prostate cancer disease; for this reason, in the next future, genomic re-assessment of the disease with a new fresh biopsy or using isolated circulating cells or circulating DNA will become desirable to personalize the therapeutic approach.

Mateo J et al. retrospectively reviewed the clinical outcome of mPC patients with and without germline DNA damage repair gene mutation (gDDRm); medical records were reviewed for 390 mPC patients with known gDDRm status. Data suggested that mPC patients with inherited mutations in DDR genes, including those with BRCA2 mutations, can achive similar benefit from standard of care therapies in terms of both response rate and PFS compared to patients without mutations [38].

Additional clinical trials are testing efficacy and safety of PARP inhibitors in combination with chemotherapy, radiotherapy or biological agents in several disease setting, including the localized disease. Phase II clinical trials are evaluating the combination of olaparib with abiraterone vs. placebo in mCRCP (NCT01972217), the combination of veliparib with abiraterone vs. abiraterone (NCT01576172) or the association of niraparib with enzalutamide (NCT02500901).

Another study evaluated the combination of veliparib (ABT-888) plus temozolamide in 26 mCRPC patients pre-treated with docetaxel: the authors demonstrated a very modest efficacy of the combination therapy with 12% of the patients achieving a PSA response >30% within 3 months (Hussain et al., 2014). Median PFS and OS were 9 weeks and 39.6 weeks, respectively. Hematological toxicities were observed; in particular, grade III/IV thrombocytopenia was noted in 15% of the patients. Despite the promising preclinical activity, this combination demonstrated disappointing results. The authors suggest that the administration of a low, sub-optimal dose of veliparib in this trial could explain the limited activity.

As observed in BRCA carriers patients affected by breast or ovarian cancer (Ahn et al., 1997; Yang et al., 2011), also carriers of mutations in DNA repair pathways could benefit from platinum-based chemotherapy and recent observations seem to support this hypothesis.

Cheng and colleagues reported some cases of very good response (complete or partial response) to platinum chemotherapy in advanced prostate cancer (Cheng et al., 2016). Retrospective DNA sequencing of these patients demonstrated a biallelic inactivation of BRCA2.

Additionaly, the retrospective multicentre analysis from Pomerantz et al confirmed that mCRPC carriers of BRCA2 mutations have a higher likelihood of positive response to carboplatin-based chemotherapy than non-carriers (Pomerantz et al., 2017). These authors retrospectively assessed a cohort of 141 mCRPC patients treated with carboplatin and docetaxel and found that 75% of the 8 BRCA2 carriers showed a PSA decline >50% compared with 17% of the 133 non-carriers.

It is clear that the studies evaluating the efficacy of platinum-based therapies in selected subgroup of patients with defects in DNA-repair genes are necessary, but the detection of mutations in DNA-repair genes could represent a predictive biomarker able to drive the therapeutic strategy. Despite the number of agents efficacious in mCRPC patients, today no robust available biomarkers are able to predict the response to a specific class of agents. Growing retrospective data could suggest a reduced activity of new hormone agents compared to chemotherapeutic agents in presence of the splice variant of androgen receptor AR-V7 (Antonarakis et al., 2014, 2015; Scher et al., 2016). Unfortunately, to date, the expression of this biomarker has only a prognostic value, since only prospective randomized trials will be able to assess its predictive value.

## Conclusions

Carcinogenesis is mediated by the accumulation of inherited or acquired genetic aberrations that promote the tumor growth advantage. DNA-repair defects can lead to an increase in genetic changes in cells resulting in an improved risk of developing cancer. The identification of the carriers of these genomic aberrations allows not only to identify people who have cancer susceptibility but also to define cancer subtypes with a different sensitivity to the treatments. It is likely that DNA sequencing will change the therapeutic approach to prostate cancer in the next years, improving molecular classification of this tumor and therefore the personalized therapeutic approach. Molecular characterization of prostate cancer seems to be promising to define also cancer prognosis.

In order to maximize the efficacy of cancer therapies avoiding unnecessary side effects, identification and prospective validation of predictive biomarkers are strongly advocated. In this context there is the need of carefully designed clinical trials which will be able to guide the tailored therapeutic approach and thus the clinical decision making process.

## Author contributions

All authors listed have made a substantial, direct and intellectual contribution to the work, and approved it for publication.

### Conflict of interest statement

The authors declare that the research was conducted in the absence of any commercial or financial relationships that could be construed as a potential conflict of interest.
